# Reply: 3B circumscribed masses: to assess or not to assess?

**DOI:** 10.1038/sj.bjc.6604535

**Published:** 2008-08-05

**Authors:** G Farshid, P Downey, P G Gill, S Pieterse

**Affiliations:** 1 Division of Tissue Pathology, Institute of Medical and Veterinary Science, BreastScreen SA, 1 Goodwood Road, Wayville, South Australia 5065, Australia; 2 Institute of Medical and Veterinary Science, Frome Road, Adelaide, South Australia

**Sir,**

We thank Drs Bonetti and Manfrin for sharing the results of their screening programme in Verona with us. The similarity in the designs of our respective programmes affords a valuable learning opportunity.

Based on their experience with 388 circumscribed 3B screen-detected mass lesions, Drs Bonetti and Manfrin confirm our data regarding the value of fine-needle aspiration biopsy as a first-line diagnostic modality for this specific category of lesions. They also subscribe to a tiered approach for the assessment of these lesions, reserving core biopsies for cases other than benign cytology.

The proportion of malignancies presenting as 3B masses in the Verona Programme is reported as 4.9%. Given the smaller numbers in their series, 388 cases compared with 1183 in the South Australian series, these findings are broadly comparable with the 8.3% rate of malignant diagnoses in our data (Farshid *et al*, 2008).

However, our recall rate is 6.7% for the first round and 2.2% for subsequent rounds, whereas they have a recall rate of 10.9% for the first round of screening and 5.4% for subsequent rounds. They point out that these rates are ‘not acceptable according to the European guidelines’ and go on to suggest that because a survival benefit has not been specifically established for cancers that present as circumscribed masses, perhaps ‘assessment of 3B lesions should be discouraged’. They then express likely reluctance in adopting this approach by clinicians, their claims managers and the clients themselves.

In essence, Drs Bonetti and Manfrin highlight the challenging balance in achieving acceptably low recall rates for breast cancer screening programmes while demonstrating a public health benefit from the exercise.

As highlighted in Table 1, there is some variation in recall rates deemed acceptable by various screening programmes and within each jurisdiction observed recall rates may well exceed the desired thresholds, sometimes by significant margins.

As 3B masses are common and mostly represent benign lesions, it is true that deferring the assessment of these lesions by adopting the policy of surveillance will reduce recall rates. However, 8.3% of these women whose lesions are indeed malignant will be denied the opportunity for early diagnosis. We therefore do not support this approach.

Quite apart from the public health and medical objections to the watchful waiting approach is the irony that this strategy will not solve the accreditation problems of programmes significantly. This is because programmes adopting this policy will potentially have to deal with substantially increased numbers of interval cancers, so that as is often the case, a measure undertaken to rectify one performance index results in the deterioration of another statistic.

In the light of the low proportion of high-grade cancers in their cohort, our colleagues in Verona raise the second issue of a survival benefit associated with the detection of cancers that present as 3B lesions. As a result of multiple randomised clinical trials, case–control series, meta-analyses and expert reviews undertaken by the IARC, there is now a high level of concordance that screening mammography is effective in reducing mortality from breast cancer. The magnitude of this effect is estimated to be in the region of 25–35% (WHO and IARC, 2002). This mortality reduction is achieved even though the majority of screen-detected cancers in these trials were of grade I or II, and indeed data from the Swedish two county trial demonstrate that it is predominantly the effects of small tumour diameter and node negativity that substantially improve survival expectations (Tabar *et al*, 2000). A recent case-controlled study evaluating our screening programme has demonstrated a 41% survival advantage for women who are regular participants (Roder *et al*, 2008). The breakdown of tumour grade in our programme is that 43.2% are grade I, 42.6% grade II and 14.3% grade III (unpublished data).

Drs Bonetti and Manfrin correctly point out that there is no definite proof that the detection of cancers that present as 3B masses will result in a reduction in breast cancer mortality. Such evidence is unlikely to be forthcoming in the form of randomised controlled trial data. The trials having proven the efficacy of screening mammography as a whole are unlikely to be repeated for each different radiologic presentation of breast cancer.

In our view, the importance of 3B mass lesions is two-fold: first, 8.3% of these lesions represent malignancies, whose detection and management will provide valuable opportunities for interrupting the natural history of some breast cancers. Second, because 3B masses are common and mostly represent benign lesions, particular care is required to reduce the physical and psychologic morbidity associated with their assessment. Our preferred approach is to utilise the least invasive methods available for their assessment, so that the cancers may be diagnosed and women with benign lesions may be reassured and return for re-screening at the correct interval.

Our contribution in this work has been the substantiation of the fact that a tiered approach using fine-needle aspiration biopsy and core biopsy achieves these outcomes successfully. A final observation is that this approach may be more cost-effective than short-term recall and repeat imaging.

## Figures and Tables

**Table 1:** Acceptable recall rates for screening mammography in different programmes

**Programme**	**First screening round**	**Subsequent screening rounds**
Australian National Accreditation Standards (2004)	<10%	<5%
United Kingdom (NHSBSP, 2005)	<10%	<7%
European guidelines (2006)	<7%	<5%
